# Physical Activity and Nutrition INfluences In ageing (PANINI): consortium mission statement

**DOI:** 10.1007/s40520-017-0823-7

**Published:** 2017-09-01

**Authors:** Anna C. Whittaker, Massimo Delledonne, Taija Finni, Paolo Garagnani, Carolyn Greig, Victor Kallen, Katja Kokko, Janet Lord, Andrea B. Maier, Carel G. M. Meskers, Nadine Correia Santos, Sarianna Sipila, Janice L. Thompson, Natal van Riel

**Affiliations:** 1 School of Sport, Exercise and Rehabilitation Sciences, University of Birmingham, Birmingham, UK; 2 Personal Genomics, University of Verona, Verona, Italy; 3 Neuromuscular Research Center, Faculty of Sport and Health Sciences, University of Jyväskylä, Jyväskylä, Finland; 4 Department of Experimental, Diagnostic, and Specialty Medicine (DIMES), University of Bologna, Bologna, Italy; 5 MRC-Arthritis Research UK Centre for Musculoskeletal Ageing Research, University of Birmingham, Birmingham, UK; 6 The Netherlands Organisation for Applied Scientific Research, The Hague, The Netherlands; 7 Gerontology Research Center, University of Jyvaskyla, Jyväskylä, Finland; 8 Institute of Inflammation and Ageing, Medical School, University of Birmingham, Birmingham, UK; 9 Department of Human Movement Sciences, MOVE Research Institute Amsterdam VU University Amsterdam, Amsterdam, The Netherlands; 10 Department of Medicine and Aged Care, Royal Melbourne Hospital, University of Melbourne, Melbourne, Australia; 11 School of Medicine, University of Minho, Braga, Portugal; 12 Department of Biomedical Engineering, Eindhoven University of Technology, Eindhoven, The Netherlands

**Keywords:** Ageing, Biomarkers, Multidisciplinary, Nutrition, Physical activity, Standardised measures


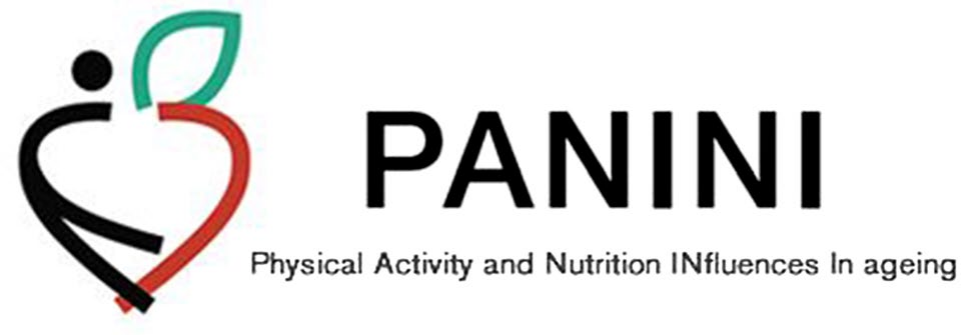


## Introduction

Current demographic trends indicate that by the year 2020, almost one in five of the European population will be aged 65 years or over. Although life expectancy is increasing by 2 years per decade, the period of life spent in good health is not keeping pace and most Europeans spend their last decade in poor health. Consequently, there is an urgent need to understand how lifestyle factors can influence age-related changes from gene to society level and how they may be integrated into a net effect of healthy ageing. It is also crucial to develop and validate interventions and health policies to ensure that more of our older adults have a healthy and active later life. This is an urgent and cross-cutting research priority in Europe, and to achieve this, it is vital to increase research capacity in this area to push forward the frontiers of scientific understanding. The Horizon 2020 funded Marie Curie Sklodowska Innovative Training Network—PANINI is addressing this capacity issue by focusing on research and training in two major interacting lifestyle factors with impact at multiple levels, namely, physical activity and nutrition.

The aims of PANINI are, therefore, to:

1. stimulate collaborative ageing research across Europe from the basic science to clinical intervention on the interaction of physical activity and nutrition through training a network of early stage researchers;
2. develop a standardised toolkit of the best practice measures of physical activity and nutrition;
3. utilise the toolkit across PANINI research projects to develop a shared data set across different European ageing populations;
4. coordinate existing data from collaborators to strengthen the PANINI data set to assess the physical activity and nutritional status interactions in a range of older adult populations;
5. compare novel physical activity and nutritional interventions to improve healthy ageing and assess the potential mechanisms underlying intervention responsiveness;
6. develop an online repository of training material on physical activity and nutrition in older adults for continuing professional development (CPD);
7. create a healthy ageing policy document with key stakeholders.

To promote healthy ageing, it is important to understand the mechanisms underlying unhealthy ageing trajectories. Healthy ageing is defined as optimising opportunities for good health, so that older people can take an active part in society and enjoy an independent and high quality of life (http://www.healthyageing.eu). Ageing is a complex process, but we are beginning to understand how age-related changes in physiological systems influence physical function. Good musculoskeletal function is critical for a healthy and active old age [1], but the musculoskeletal system is significantly affected by ageing, with loss of muscle mass and bone density from midlife resulting in increased physical frailty, falls, fractures, and loss of independence. The aetiology of sarcopaenia (low skeletal muscle mass and function) includes intrinsic factors associated with ageing, but more recently extrinsic modifiable lifestyle factors such as physical activity and nutrition have been suggested as primary drivers [2]. When associated with weight loss, fatigue, weakness, slow walking speed, and physical inactivity, this results in frailty [3]. Thus, early detection through comprehensive multidisciplinary assessment of a range of relevant factors and sensitive biomarkers, and mitigation through targeted interventions is essential [3].

Increased inactivity is a well-recorded feature of ageing humans, with less than half of those over the age of 50 years meeting the recommended guidelines for physical activity, and less than 10% in those over the age of 75 years. Furthermore, ageing is typically associated with a gain in fat mass (adiposity), which with decreased activity can contribute to sarcopaenia, and reduced mobility [4]. However, with ageing, energy intake also decreases, and malnutrition is prevalent [5], particularly in hospitalised patients and older outpatients [6]. Physical activity and optimal nutrition can influence the function of a range of body systems [7, 8], which interact in the maintenance of homeostasis and healthy ageing via enhancing quality of life, cognition, muscle mass, decrease fat mass, and modify sarcopaenia. However, data are sparse on energy-protein intake and energy expenditure in less fit older populations or community-dwelling older adults, where the prevalence of undernutrition may be far higher than currently assumed [7]. Consequently, nutritional and exercise requirements of elders to maintain physical and mental function are largely unknown, although the European Society for Clinical Nutrition and Metabolism (ESPEN) [9] have made evidence-based nutrition recommendations and the European College of Sport Science (ECSS) [10] have adapted existing physical activity guidelines for older adults. What is needed are multidimensional data over various populations, extending from mid-adulthood to old age, which PANINI will collect and analyse through development of its shared database and standardised toolkit of measures detailed below.

Understanding the biology and physiology underlying healthy/unhealthy ageing is an important initial step required before developing and testing physical activity and nutritional interventions. PANINI will address this through focusing on the impact of nutrition and physical activity on mobility, muscle function, endocrine function, cognitive function, and inflammation in ageing at the cell and system level. Dysregulation of mitochondrial function in the muscle is a hallmark of ageing and age- and lifestyle-related diseases and so will be studied in skeletal muscle, applying a system biology approach through the mathematical modelling of metabolism [11]. This will include assessment of the impact of diet and physical activity on metabolic activity across different age groups through the use of the newly generated PANINI data as well as existing data sets of PANINI collaborators, e.g., NU-AGE, MYOAGE, and MUMC+.

Stress hormone changes with ageing relate to significant decrements in mental health and frailty, and increased inflammation, thus cortisol:dehydroepiandrosterone (DHEA) hormone ratios will be measured [12]. The decline in sex steroids in perimenopause can also have dramatic effects on muscle mass and function later on in life [13]. However, few studies have characterized changes in neuromuscular function, level of physical activity, and psychological characteristics in middle-aged women across menopausal stages. These biological and physiological changes will be studied within PANINI. Biological and physiological changes associated with ageing will be examined across a range of older populations of differing frailty phenotype, and novel interventions will be designed to reduce sedentary time, increase activity, and improve physical function. The study of sedentariness, and the development of interventions to reduce this will focus on older adults awaiting surgery and will utilise behaviour change techniques such as individualised feedback, individualised goal setting, and motivational interviewing, as well as environmental modification to encourage reduced sitting time. It will also utilise direct measurement of physical activity and sitting time variables to provide data on sitting time, standing time, stepping time, sit-to-stand transitions, and quantity of sedentary bouts >30 min. These data will be used to inform the design of a definitive trial to reduce sitting time prior to surgery, on selected health outcomes after surgery.

Further, ageing is often accompanied by an increase in systemic inflammation affecting physical and mental function [14], and dietary intake can also influence memory, attention, and learning [15]. Consequently, where possible within PANINI, cognitive function will also be assessed, and in particular, elderly cohorts, detailed food intake, and cognitive function will be assessed longitudinally to assess the impact of dietary choices and changes on cognitive decline. In addition, assessing dietary intake is challenging and subject to bias, but quantitative methods are cost- and labour intensive and only provide a narrow view of nutrients. PANINI will also use a mixed-methods approach that can provide a richer and broader view of food choice and eating behaviours. We will focus specifically on changes in social networks and their influence on dietary intake and physical function and will target under-studied, high-risk groups such as ethnic minorities, allowing us to design appropriate interventions that address barriers and enablers to sustained healthy food choices in old age across a range of populations.

The state of the art for interventions to improve musculoskeletal health is resistance training with/without nutritional supplementation [16], but efficacy is blunted in older adults, so innovative strategies to optimise responsiveness are needed. Physical activity interventions need to be appropriate for older adults across a range of health, frailty, and independence levels, and, as such, must be individually tailored. For example, major surgery induces stress, which combined with bed rest, can be harmful for patients, particularly when frail. Promoting a pro-physical activity and social environment before and during the entire surgical trajectory prevents frailty progression, and has been proven cost effective [17]. One of the barriers to physical activity in frail or recuperating populations is limited mobility and independence. There is evidence to support the effectiveness of chair-based muscle strengthening resistance exercise on muscle function [18]; however, it is not yet known how feasible these interventions are in very frail and dependent older adults, or how effective they are at altering key biomarkers of healthy ageing and well-being such as inflammation. PANINI will refine existing interventions by determining feasibility, effectiveness, and appropriate dose in a range of frail older adult populations including acute inpatients, sedentary older adults, care home residents, and hospital outpatients.

Discovering biomarkers of ageing are a crucial goal in ageing research. Epigenetics are the range of heritable reversible changes in chromatin structure and gene function, which can determine biological ageing and health status. Several candidate genes have been identified that show progressive increases in methylation across the human lifespan, and thus appear to be promising biomarkers [19, 20]. However, further research is needed to determine whether gene methylation simply indicates chronological age or correlates with physiological function. Using this state-of-the-art technique, it is possible to develop interventions that could alter biological ageing via effects on epigenetic regulators, such as in FP7 project NU-AGE [21], focusing on the modulatory role of a modified Mediterranean diet on DNA methylation patterns during ageing [21, 22]. PANINI will expand this research by also examining methylation of candidate genes that relate to physiological function, and assess the impact of interventions on epigenetic modulation to provide further information on mechanisms of intervention effectiveness across several of the PANINI interventions. DNA methylation analysis of candidate biomarkers will be performed using Sequenom MassArray high-throughput platform and quantitatively analysed for DNA methylation using the EpiTYPER protocol. Candidate target regions will include genomic regions, whose methylation status are age-associated and are informative of the biological age of an individual, according to published and proprietary data.

Finally, PANINI will explore the relevance of individual genetic background for the development of sarcopenic/frail phenotypes and for the success of nutritional and physical interventions. Studies of genetic determinants of muscle mass/strength have revealed different risk phenotypes for sarcopaenia [23–25]; however, an under-researched but relevant field is the relationship between nutritional habits and variants in genes that affect appetite and dietary preferences. PANINI will address this through comprehensively studying the interplay between genetic background, diet and sarcopenic/frail phenotype. Genetic analysis will stratify participants for their response to physical and nutritional interventions across PANINI. Next-generation sequencing (on a sub-sample) will integrate these results with the genetic characterizations in NU-AGE; candidate genomic variants will be evaluated on a larger mixed gender cohort. Metabolic network modelling will be used to study changes in energy metabolism associated with ageing. Model predictions will be validated with molecular data from a select number of participants in the PANINI data set. The main projects in PANINI and their key methodologies and outcome variables are summarised in Table 1. Publications as they arise will be open access and linked from the PANINI website http://www.birmingham.ac.uk/panini.

**Table 1:** Summary of main projects in the PANINI network

ESR and location	Projects	Main methodologies	Sample/cohort	Primary outcome variables
1. Vrije University, Amsterdam, and University of Birmingham	Development of PANINI standardised toolkit Creation of PANINI shared data set	Assessment of prevalence of malnutrition and low physical performance/activity according to different risk screening and assessment tools over different ageing populations in Europe	New PANINI data and existing data sets (Birmingham Hip Fracture, EMPOWER, MUMC+, COGA, ERMA, Grey Power 1 and 2, NU-AGE, ProMo and Bronovo)	Mini Nutritional Assessment (MNA), Short Nutritional Assessment Questionnaire (SNAQ), hand-grip strength, Short Physical Performance Battery (SPPB), Timed up and go, and gait speed
2. University of Birmingham	Seated Physical Activity in Ageing (SPAA) feasibility study Keeping Active in Residential Elderly (KARE) feasibility study	Resistance training using HUR leg strength training machine versus Move it or Lose it activity programme Resistance training using HUR strength training machines	Hospitalised frail older adults Frail residents of care homes aged 60+ years	Feasibility, Physical function (SPPB, hand-grip strength), Blood measures (CRP, IL-6, TNFa, Cortisol, DHEA/S)
3. University of Birmingham	Intervention to reduce sitting time in older adults undergoing orthopaedic surgery: a feasibility study Protocol for a definitive trial	Behaviour change techniques (individualised feedback, individualised goal setting, motivational interviewing, phone calls, health education, environmental modification)	Orthopaedic patients aged 65 years and over	Feasibility, Physical activity—IPAQ & ActivPal accelerometer, Measure of Older Adults’ Sedentary Time (MOST), Blood measures (Albumin, HDL, LDL, triglycerides, Vit. D., CRP, IL-6, TNFa, Cortisol, DHEA/S, Transferrin)
4. University of Birmingham	Diet and eating behaviours in ethnically diverse older adults	Explore changes in social networks over 8-months and assess their prediction of dietary intake and physical function via the use of the Wenger Social Network Typology tool, repeated 24-hr recalls with in-depth semi-structured interviews	Community-dwelling ethnically diverse adults aged 60 years and older	Dietary intake, influences on eating behaviours, social network typology, physical function (SPPB)
5. Eindhoven University of Technology	Ageing and lifestyle impact on skeletal muscle function	Systems Biology, computational modelling, genome-scale metabolic models (GSMM), machine learning	New PANINI data, NU-AGE, MYOage, MUMC+	Quantitative and predictive models, describing metabolic activity (flux) in different age groups, predicting effects of diet and physical activity
6. University of Minho, Portugal	Nutrition effects on well-being and cognitive function	Collection of dietary data of ‘good’ and ‘poor’ cognitive performers and ascertainment of the impact of dietary variables on cognitive trajectories. Study of the impact of dietary changes on cognitive performance	Community-dwellers aged 55+ years	Neuropsychological evaluation Nutritional intake (food diaries, food frequency questionnaires)
7. University of Jyvaskyla, Finland	Physical activity, muscle function and psychological characteristics during menopausal transition	Comprehensive assessment of muscle function, physical activity and psychological characteristics in middle-aged women carefully characterized according to the menopausal status. Cross-sectional and follow-up designs	48–55 year old women characterized, according to the bleeding diary and hormonal assessment, as being pre-, peri- or postmenopausal	Muscle function; hand-grip and knee extension strength, muscle power, gait speed (6 min and 10 m). Psychological characteristics: depression and life satisfaction
8. The Netherlands Organisation for Applied Scientific Research	Mechanisms of physical exercise effects in surgical patients	Preoperative risk stratification, assessment of physical fitness, (home-based) prehabilitation high-risk patients	Patients undergoing major elective abdominal surgery	Feasibility, steep ramp test performance, hand-grip strength, timed up-and-go test, five times sit-to-stand test, time to recovery of physical functioning, morbidity, length of stay
9. Vrije University Amsterdam	Nutrition and physical activity to counteract sarcopaenia	DEXA, BIA, food frequency questionnaire, ventilated hood	SHAPE study (geriatric outpatients with fall risk)	Muscle mass
10. University of Bologna	Epigenetics of nutrition in ageing	DNA methylation analysis (gene-targeted) by Sequenom ® MassARRAY EpiTYPER platform	NU-AGE samples and new PANINI data	Changes in DNA methylation patterns induced by dietary and physical interventions. Development of a gene-targeted epigenetic clock
11. Personal genomics	Genetics of nutrition in ageing	Development of a bioinformatic pipeline for the identification of Single Nucleotide Variations correlated with ageing phenotypes and response to nutritional interventions of elders. Common annotation sources will be exploited to integrate all the information available for a single variant	NU-AGE and PANINI data	List of genetic variants correlated with ageing phenotypes and nutritional interventions

NB all projects will implement the PANINI toolkit, but some parts may be secondary outcome variables depending on the research question

## The PANINI toolkit and shared data set

PANINI will recommend a key set of gold standard nutrition and physical activity measures in ageing as a standardised toolkit, and make this widely available outside the PANINI consortium to encourage standardised measurement. Through the toolkit, we will bring together all of the PANINI projects to contribute to the development of a shared database for analysis of various ageing profiles across the range of older adult populations. Our data management plan (available on the PANINI website) is that this standardisation will result in a unique shared data set from which ageing profiles across countries, settings, frailty, and independence status could be determined and compared focusing particularly on nutritional status, physical activity, and physical function, as well as comparing relative change across the interventions in the network. The main components of the toolkit are: socio-demographics; anthropometrics (including body composition where possible); comorbidities and medication use; typical health behaviours including water intake; nutritional assessment questionnaire (and food frequency questionnaires or food/diet diaries where possible); physical activity via questionnaires (and accelerometry where possible); physical function via the short physical performance battery (SPPB); frailty through hand-grip strength, balance, and walking speed; falls efficacy and activities of daily living scales; brief cognitive assessment; and depression assessment.

We will also link, where possible, with matching data from existing European cohorts on ageing. For example, through the beneficiaries of the network and their existing links, we will collaborate to examine existing nutritional and physical function data in the Birmingham Hip Fracture, EMPOWER, MUMC+, COGA, ERMA, Grey Power 1 and 2, NU-AGE, ProMo, and Bronovo studies, with appropriate permissions. The PANINI shared data set will be made an open access resource at the conclusion of the project enabling the PANINI consortium and collaborators to answer research questions regarding physical activity and nutritional status impacts on healthy/unhealthy ageing. Researchers or networks wishing to become affiliated with the PANINI network for collaboration and/or data contributions to the shared data set should contact the Principal Investigator (PI)—Professor A. C. Whittaker.

## PANINI training

To understand the impact of age-related changes upon normal body processes such as nutritional intake, and exercise capacity, and to relate this to overall effects on physical and mental well-being, a multidisciplinary approach is required. PANINI is providing multidisciplinary PhD training and secondments (summarised in Table 2) across an inter-sectoral network of eight leading academic and non-academic research institutions across Europe (six universities, one research enterprise, and one SME, as detailed in Table 1). Our partners: six private sector companies (Danone Nutricia Research; HUR Ltd.; HURLabs; Move it or Lose it; Scriptoria; and Blueberry Training); one large healthcare partner (University Hospitals Birmingham NHS Foundation Trust); two ageing charities (Age UK and UNIEKBO); and one Joint Research Council, who contribute to training, supervision, and dissemination, providing an innovative and inter-sectoral environment to produce maximum impact of PANINI’s research. We are keen to open up the PANINI training network to other research and training networks; our main activities, open to (a limited number of) external participants, are detailed in Table 2; so please contact the PI if interested. It is also our plan to develop a CPD distance learning course from the materials developed and delivered during the PANINI project, after its completion, to increase training resources and build research capacity in multidisciplinary ageing research.

**Table 2:** Main PANINI activities

Activity	Month (from Jan 2016)
Kick-off meeting	2
Launch PANINI website and social media	3
Data management plan finalised	6
Standardised measures toolkit developed	9
Standardisation: Shared database set up	14
ATC1: Biology of Ageing; Theories of Ageing; Musculoskeletal ageing; Brain ageing and cognition; Obesity, metabolism and ageing	14*
ATC2: Physical activity and ageing; Sarcopaenia and exercise effects on muscle; Physical activity interventions for healthy ageing	14*
ATC3: Nutrition and ageing; Genetics and longevity; Nutritional intake and protein-energy balance; Dietary patterns and ethnic and cultural variations	14*
Network meeting: ESR Project presentations	14*
ESRs individual public engagement projects	Annually
ATC4: Outreach & dissemination. Scientific communication for non-specialists (Scriptoria)	14*
Group Secondment 1: Healthcare setting (University Hospitals Birmingham) New hospital design; Older people in Hospital: Reminiscence, Design, Feedback, Listening to patients; Older people medicine: The in-patient experience, Treating the older person; Nutrition: General health and well-being, Eating to stay healthy, Nutritional care after trauma, Medication and nutrition; Post Stroke Care: The swallow reflex, Speech, Facial asymmetry, Mobility; Falls: Prevention of harm, Fracture management, Rehabilitation, Returning home	25
Public engagement event: Stakeholder involvement	25*
ATC5: Translation of research into product – (1) Physical activity research into intervention product development (Move it or lose it); (2) How to translate genetic/epigenetic biomarkers research into a commercial tool (Personal Genomics)	25*
Visiting Scientist visit	25*
Network meeting: ESR Project presentations	25*
ATC6: Business engagement. Formulating a business model towards a sustainable products and services portfolio; Formulating a business case for innovations; Enhancing readiness towards investors and venture capital; Protecting the IP portfolio (TNO)	35*
ATC7: Enterprise training. Creativity & enterprise skills and the translation of research activity into products, patents and spin-off companies (Blueberry Training)	35*
Group Secondment 2: Industry and technology. technology development and innovation for physical function assessment (HUR & HURLabs); technology development for nutrition delivery to vulnerable older adults (Danone Nutricia Research); training and experience in innovation in patients’ self-health assessments & the Better in Better out concept (TNO)	35
ESR podcasts disseminated	42
PANINI symposia at interdisciplinary international conference(s) – dissemination of scientific progress	By 42
Network conference	44*
ATC8: Getting research into health policy. Health Policy Legislation by the Healthy Ageing Commission Chair and UK Chief Inspector of GPs; EU legislative framework: how policy works at EU level; EC vs. member states competencies in health policy making (Age UK, UNIEKBO)	44*
ATC9: Health Policy document development - Introduction on the policy cycle (PC): To discuss how research is needed in each phase of the PC; Case study: How does the JRC support the EC in policy making? Turning research documents into policy-ready information; Discussion: How will your research support policy making in your member state? (JRC)	44
Network outreach and dissemination event	44*
Develop CPD distance learning course based on PANINI materials	45 onwards
Make PANINI toolkit and shared database open access	56 onwards

*ATC* advanced training course, *ESR* early stage researcher

* Limited spaces open to other researchers/networks, contact the PI A.C. Whittaker

## Outreach, dissemination, and impact

PANINI’s approach to dissemination, impact, and innovation is to co-create our strategy with our stakeholders, holding public engagement events annually. These will allow us to refine our research methodology and dissemination approaches to meet stakeholder needs and expectations. Researchers will engage the public via annual individual or small group interactive activities such as science association events, older adults’ societies, articles in older adults’ newsletters, pod/vodcasts, and the media in a widely accessible manner. Further, an initial PANINI public engagement event is planned for midway through the project, where the ESRs will engage older adults with key messages and demonstrations from their projects. A final larger scale public engagement event is planned for the end of the network. Older adults and representatives from charities, industry, and the health sector with an interest in ageing research will be invited to attend these events. Midway through the project, it is our intention to present PANINI symposia at relevant interdisciplinary conferences with a focus on ageing, presenting the goals of the network and initial findings from the individual ESR projects and their collaborative work. Towards the end of the project, with key stakeholders, e.g., ageing charities, health professionals, and policy makers, we will refine a set of physical activity and nutritional intake recommendations for older adults of different frailty and dependency statuses which will be integrated with the knowledge gained regarding nutritional intake and physical function across the network. This will be integrated into a policy document for healthy ageing and discussed with representatives of health policy-making bodies at national (e.g., Public Health England) and European levels (e.g., EIP-AHA and DG-SANCO). These activities are summarised in Table 2; our dissemination plan is available on the PANINI website.

In conclusion, a key target of PANINI is to develop a cohort of the next-generation researchers able to communicate healthy ageing research across disciplinary boundaries and value the benefits of the cross fertilisation of ideas and problem solving across the emerging interdisciplinary areas essential for integrative ‘whole person’ physical activity and nutrition research. Key impact will be through the anticipated widely disseminated significant advances in understanding the central processes that contribute to healthy ageing and the factors and mechanisms underpinning successful interventions. Key outputs will be a PANINI toolkit of recommended measures; a shared data set used to interrogate health and well-being in ageing across a range of older populations as predicted by the toolkit and a co-created health policy statement. Further, the training resources will also be made widely available in order as a resource to develop research capacity in physical activity and nutrition in ageing.
